# Supplementary material for: High-throughput spatial sensitive quantitative phase microscopy using low spatial and high temporal coherent illumination
Source: Sci Rep. 2021 Aug 4;11:15850. doi: 10.1038/s41598-021-94915-w (PMC8338969; doi:10.1038/s41598-021-94915-w)
Supplement: Supplementary file 1 — Supplementary Information. [file 41598_2021_94915_MOESM1_ESM.docx]

**Supplementary Information**

**High-throughput spatial sensitive quantitative phase microscopy using low spatial and high temporal coherent illumination**

Azeem Ahmad^1,*^, Vishesh Dubey^1^, Nikhil Jayakumar^1^, Anowarul Habib^1^, Ankit Butola^1^, Mona Nystad^2^, Ganesh Acharya^2,3^, Purusotam Basnet^2^, Dalip Singh Mehta^4^, Balpreet Singh Ahluwalia^1,3,*^

^1^Department of Physics and Technology, UiT The Arctic University of Norway, Tromsø 9037, Norway

^2^Department of Clinical Medicine, Women’s Health and Perinatology Research Group, Faculty of Health Sciences, UiT - The Arctic University of Norway, Tromsø, Norway and Department of Obstetrics and Gynecology, University Hospital of North Norway, Tromsø 9037, Norway

^3^Department of Clinical Science, Intervention and Technology Karolinska Institute, and Center for Fetal Medicine, Karolinska University Hospital, Stockholm 17177, Sweden

^4^Applied Optics and Biophotonics Laboratory, Department of Physics, Indian Institute of Technology Delhi, New Delhi 110016, India

Email: [ahmadazeem870@gmail.com](mailto:ahmadazeem870@gmail.com) and [balpreet.singh.ahluwalia@uit.no](mailto:balpreet.singh.ahluwalia@uit.no)

**Video 1:** Interferometric movie of live human sperm cells.

**Video 2:** Reconstructed high-resolution phase movie of the sperm cells.

1. **Effect of rotating diffuser speed on spatial phase sensitivity:**

Figure S1 illustrates the spatial phase noise of QPM system for PTLS as a function of the rotating diffuser speed in revolutions per minute (RPM). Table S1 depicts the values of spatial phase noise as a function diffuser RPM. It can be clearly visualized from the measured values of phase noise that the speed of the diffuser does not influence the phase noise of the system significantly if it is greater than or equal to 120 RPM. The phase noise is measured to be equal to 3.4 mrad for the diffuser speed from 120 RPM to 1500 RPM. The diffuser speed of 90 RPM increased the phase noise slightly and measured to be equal to 3.7 mrad. We could not perform experiments with the diffuser speed less than 90 RPM due to lower speed limit of our DC motor. However, we believe that the diffuser speed less than 90 RPM would further increase the phase noise of the system due to insufficient averaging of speckle patterns.

**Fig. S1.** Spatial phase noise variation of QPM system as a function of rotating diffuser speed in RPM. It exhibits the histogram plot of the measured phase noise for different RPM values of the diffuser.

**Table S1:** Spatial phase noise variation of QPM system as a function of rotating diffuser speed in terms of RPM.

| **S. No.** | **Diffuser speed (RPM)** | **Phase noise (mrad.)** |
| --- | --- | --- |
|  | 90 | 3.7 |
|  | 120 | 3.4 |
|  | 240 | 3.4 |
|  | 700 | 3.4 |
|  | 920 | 3.4 |
|  | 1500 | 3.4 |

1. **Static interference formation under unbalanced optical configuration:**

Thanks to the reflection geometry of the present QPM system, the speckle fields passing through different objective lenses in the object arm remains unchanged and correlated. To understand the phenomenon behind this, first, the speckle sizes of the fields coming from the object arm only is calculated for both objective lenses. The average speckle sizes of the patterns shown in Figs. S2 are given in Table S2. It can be seen that the average speckle size does not change much for both objective lenses. This is due to the reflection geometry of the present phase microscope, the speckle field coming from the MMF (Fig. 8) is passing twice through the same objective lenses in both object and reference arms. The objective lens, first, de-magnifies the speckle size of the input field and forms diffraction limited speckles at the sample and reference mirror. The same objective lenses collect the speckle fields reflected from the sample and the reference mirror. The objective lenses again magnify the demagnified speckles by the same amount. Thus, the speckle size remains unchanged and correlated while passing through different objective lenses in the reflection geometry.

Figures S2 illustrates the experimental outcomes for both objective lenses 20×/0.45NA and 60×/1.2NA. The object ‘O’ and reference ‘R’ arm speckle fields corresponding to 20×/0.45NA and 60×/1.2NA objective lenses are depicted in Figs. S2(a, b) and S2(f, g), respectively. The reference and the object arms are sequentially blocked to record the object and reference speckle pattern. The correlations between O and R speckle fields are then calculated using MATLAB corresponding to both objective lenses inserted in the object arm and tabulated in Table S2.

**Fig. S2.** Experimental study of the superposition of object arm and reference arm speckle fields corresponding to sequentially inserted two different objective lenses 20×/0.45NA and 60×/1.2NA in the object arm keeping 10×/0.25NA in the reference arm. (a, f) and (b, g) Represent the speckle fields coming from the reference and the object arm corresponding to 20×/0.45NA and 60×/1.2NA objective lenses. (c) and (h) 2D normalized correlation between the object and reference speckle fields. (d) and (i) Specklogram generated due to the superposition of the object and reference speckle fields at the detector. The diffuser is kept stationary. (e) and (j) Corresponding averaged interferograms recorded by the camera when the diffuser is rotated. A large number of specklograms are averaged within the exposure time of the camera. The insets represent the zoomed views of the regions marked with yellow color boxes. SD: stationary diffuser; RD: rotating diffuser; O: object arm objective lens; R: reference arm objective lens.

First, the correlation between the object and the reference arm speckle fields is calculated. The 2D normalized correlation maps are illustrated in Figs. S2(c) and S2(h), respectively. It can be clearly seen from these figures that the amounts of correlations between R and O speckle fields do not vary significantly for both objective lenses. The values of the correlation between the object and the reference arm speckle field are found to be equal to 0.66 and 0.73 for 20×/0.45NA and 60×/1.2NA objective lenses, respectively. In order to record the specklograms corresponding to both objective lenses, the object and the reference arms are opened. Figures S2(d) and S2(i) present the specklograms corresponding to 20×/0.45NA and 60×/1.2NA objective lenses, respectively. The contrast of the fringes in the specklogram is very good. The time averaged interferograms within the exposure time of the camera are illustrated in Figs. S2(e) and S2(j), respectively. Thus, the use of different objective lenses in the object arm keeping fixed objective lens in the reference arm does not destroy the correlation between their speckle fields and forms nicely oriented interference patterns.

The situation will be different in case of transmission geometry where the speckle field passes only once through the objective lens. The different objective lenses will magnify the speckle field differently and subsequently change the speckle size at the detector. Thus, in order to obtain correlated speckle fields in transmission geometry, identical objective lenses must be used in both object and reference arms. In addition, the position of the object and reference arm objective lenses from the detector plane must be equal. Slight misalignment in the object and reference arm either changes the speckle size or decorrelates the speckle fields at the detector. However, it is not necessary to use diffraction grating in the optical beam path to obtain high density interference fringes at the camera.

**Table S2:** **Speckle sizes as a function of objective lens magnification and NA**

| **S. No.** | **Objective lens** | | **Average speckle size (pixels)** | **C_OR_ (a.u.)** |
| --- | --- | --- | --- | --- |
|  | **Reference arm** | **Object arm** |  |  |
|  | 10×/0.25 | 20×/0.45 | 11.7 | 0.66 |
|  | 10×/0.25 | 60×/1.20 | 12.3 | 0.73 |

1. **Power loss study after every optical component**

In the present optical configuration, only 10 % intensity of the input beam is obtained at the input port of QPM system and rest of the 90 % intensity is lost at different optical components. The loss of power can be justified as it brings spectral purity in the light source almost equal to laser and other advantages as highlighted in the manuscript. Further, nowadays very high-power lasers are easily available in the market which can overcome the problem of intensity loss. The power of the input light beam and after every component is given as follows:

Power of the I/P laser beam: 5.0 mW

Power after rotating diffuser: 2.8 mW

Power after lens or at the I/P of multimode fiber: 2.0 mW

Power at the O/P of multimode fiber: 0.5 mW

We believe the power at the output port of multimode fiber can be further improved by employing optimized combination of fiber coupling lenses.
